# Predictive risk factors for anastomotic leakage after anterior resection of rectal cancer in elderly patients over 80 years old: an analysis of 288 consecutive patients

**DOI:** 10.1186/s12957-019-1655-z

**Published:** 2019-06-29

**Authors:** Sicheng Zhou, Haitao Zhou, Zhaoxu Zheng, Jianwei Liang, Zhixiang Zhou, Xishan Wang

**Affiliations:** Department of Colorectal Surgery, National Cancer Center/National Clinical Research Center for Cancer/Cancer Hospital, Chinese Academy of Medical Sciences and Peking Union Medical College, Beijing, 100021 China

**Keywords:** Rectal cancer, Anastomotic leakage, Risk factor, Elderly patient, Comorbidity

## Abstract

**Background:**

Anastomotic leakage (AL) is a common complication after anterior resection of rectal cancer. Few studies have been conducted to determine whether the traditional predictors of AL can be applied to elderly patients (age ≥ 80) undergoing anterior resection (AR) or low anterior resection (LAR) of rectal cancer. This study was designed to explore the predictive factors for AL after anterior resection of rectal cancer in patients over 80 years old.

**Methods:**

From January 2007 to May 2019, consecutive elderly (age ≥ 80) rectal cancer patients undergoing AR or LAR at our institution were systematically reviewed. The general information, perioperative outcomes, and comorbidities were collected.

**Results:**

A total of 288 consecutive patients were included in this study. The average age was 82.8 ± 2.4 years, and 30 (10.4%) patients developed AL. The univariate analyses showed that neoadjuvant therapy (50.0% vs. 27.9%, *P* = 0.013), the number of stapler firings ≥ 3 (60.0% vs. 36.0%, *P* = 0.011), and coronary heart disease (CHD) (46.7% vs. 17.8%, *P* < 0.001) were associated with an increased incidence of AL. The multivariate analysis showed that the number of stapler firings ≥ 3 (OR = 4.77, 95% CI = 1.33–15.21, *P* = 0.035) and CHD (OR = 8.33, 95% CI = 1.94–13.05, *P* = 0.003) were independent risk factors for AL.

**Conclusion:**

The number of stapler firings ≥ 3 and CHD were independent risk factors for AL in elderly patients (age ≥ 80) with rectal cancer. A temporary ileostomy or the Hartmann procedure is recommended for patients with CHD, male patients, patients considered to be obese, and patients with a lower tumor location, which may increase the number of stapler firings. Certainly, we recommend that the number of stapler firings should be minimized to alleviate the economic and physical burden of patients.

## Background

With decreases in fertility and mortality rates, the problem of population aging is becoming increasingly severe [1]. The risk of colorectal cancer increases exponentially with age. According to the Globocan 2012 database of the World Cancer Research Center, it is estimated that the incidence of colorectal cancer in those over 75 years old in China is approximately 78,200 every year, accounting for 18% of the global incidence of colorectal cancer [2].

Anastomotic leakage (AL) is a common complication after anterior resection for rectal cancer. It not only significantly increases local recurrence rates and reduces long-term survival rates but also extends the length of hospitalization and wastes unnecessary medical resources [3–7]. Patients with AL tend to suffer severe consequences, including peritonitis, widespread inflammation, organ failure, and septic shock. Elderly colorectal cancer patients with poor physical health, including diabetes, hypertension, coronary heart disease (CHD), and other comorbidities, as well as younger patients without these comorbidities, may not be able to cope with the physiological insult when AL occurs. A large number of studies have confirmed that gender, location of the anastomotic site, sarcopenia, preoperative albumin level, and other factors are closely related to the occurrence of AL. These factors can be used as predictive indicators of AL; however, most of them are targeted at young patients [5, 6, 8–10]. Few studies have been conducted regarding whether the traditional predictors of AL can also be applied to elderly patients undergoing anterior resection (AR) or low anterior resection (LAR) for rectal cancer. Therefore, we conducted a single-center retrospective study with a large sample size to identify the risk factors for AL in elderly patients (age ≥ 80) with rectal cancer.

## Patients and methods

From January 2007 to May 2019, a total of 6185 consecutive rectal cancer patients underwent AR or LAR at the National Cancer Center/National Clinical Research Center for Cancer/Cancer Hospital, Chinese Academy of Medical Sciences, and Peking Union Medical College. The inclusion criteria were as follows: (1) patients aged 80 or older, (2) patients with tumors less than 15 cm from the anus and pathologically confirmed as adenocarcinoma, and (3) patients who underwent open or laparoscopic AR and LAR. The exclusion criteria were as follows: (1) patients with distant metastases or extensive abdominal implantation; (2) patients who underwent emergency surgery for reasons such as intestinal obstruction, bleeding, or perforation; and (3) patients with synchronous primary tumors. Finally, 288 patients met the criteria and entered this study.

In the present study, AL was defined as damage to the integrity of the intestinal wall of the anastomotic site caused by necrosis or abscess formation, resulting in communication between the intraluminal and extraluminal compartments of the abdomen according to the definition of AL proposed by the International Study Group of Rectal Cancer (ISREC) in 2010 [11]. AL was classified into three grades: grade A—only radiological evidence of a leak, without any treatment required; grade B—a symptomatic leak that requires either antibiotics or a percutaneous drain; and grade C—a symptomatic anastomotic leak that requires return to theater and a laparotomy. AL was diagnosed by meeting the criteria for grade B or C in the present study. Pelvic drainage tubes were routinely placed in all patients, and the pelvic drain was removed when the output of the drain was clear and lower than 50 mL/24 h. Patients with delayed AL more than 1 month after surgery were excluded from this study.

We defined elderly patients as being 80 years old or older. All procedures were performed by colorectal surgeons with more than 20 years of surgical experience, and all enrolled patients underwent radical surgery in accordance with the total mesorectal excision (TME) principle. The American Joint Committee on Cancer (AJCC, the seventh edition) staging system was used for tumor staging. All patients received a preoperative assessment, including physical and laboratory examinations, colonoscopy with biopsy, abdominal computed tomography scan, and pelvic magnetic resonance imaging. The guidelines of the National Comprehensive Cancer Network were used for perioperative management. Patients with T3, T4, or N+ middle or low rectal cancer received neoadjuvant chemoradiotherapy followed by surgery 6 weeks later. All patients received the same perioperative treatment regimen and routinely underwent bowel preparation and received prophylactic antibiotics for 1 day. Written informed consent was obtained from each patient included in the study. The ethics committee of the Cancer Hospital, Chinese Academy of Medical Sciences, approved this retrospective study (NCC 2017-YZ-026, Oct 17, 2017).

### Surgical procedure

The patient was placed in a modified lithotomy position with a pneumoperitoneum of 12 mmHg. Four trocars (2 × 12 mm and 2 × 0.5 mm) were used in most cases, and surgical skill was applied according to the radical principle. High ligation of the inferior mesenteric vessel, mobilization of the bowel, and dissection of the lymph nodes were performed, and total mesorectal excision with nerve-sparing techniques was performed for rectal cancer. One or more linear staplers were used to divide the distal rectum at least 2 cm below the tumor, and the line of division was generally transverse or oblique. A small incision 3–7 cm in length was made in the hypogastrium, and transection of the rectum and mesentery was completed through an abdominal incision. An anvil head was fixed in the proximal sigmoid colon, and end-to-end colorectal anastomosis was performed with a circular stapler using the double-stapling technique for all cases. Selective temporary ileostomy was performed based on the observation of intestinal wall blood supply and experience of the surgeons.

### Statistical analysis

The Statistical Package for the Social Sciences (SPSS) version 24.0 for Windows (IBM Corp, Armonk, NY, USA) was used for data analysis. Quantitative data are expressed as the means ± standard deviations and were analyzed by Mann-Whitney *U* tests. Categorical data were analyzed by the chi-squared test or Fisher’s exact test. Multivariate logistic regression analysis was used to analyze statistically significant variables in univariate analysis, and odds ratios (ORs) and 95% confidence intervals (95% CIs) were used to assess the relationships between these factors and AL. All tests were two-sided, and a *P* value less than 0.05 was considered to indicate statistical significance.

## Results

### Characteristics of patients enrolled in this study

The clinical and pathological characteristics of all patients are shown in Table 1. A total of 288 consecutive patients with rectal cancer whose mean age was 82.8 ± 2.4 years were included in our study, including 149 (51.7%) male and 139 (48.3%) female patients. Their mean BMI was 23.9 ± 3.5 kg/m^2^. A total of 102 (35.4%), 120 (41.7%), and 66 (22.9%) patients had cancer of the lower, middle, and upper rectum, respectively. T3 and T4 stage cancers were most prevalent (61.1%), followed by T1 and T2 stages (38.9%). The mean tumor size and the length of specimens removed were 4.6 ± 1.6 cm and 10.5 ± 2.9 cm, respectively. The mean number of harvested lymph nodes was 16.9 ± 7.3, and 24 (8.3%) patients had fewer than 12 lymph nodes harvested.

**Table 1:** Univariate analysis of clinical and pathological characteristics

Factors	Anastomotic leakage (+) *n* = 30	Anastomotic leakage (−) *n* = 258	Total *n* = 288	*P*
Age (years, mean ± SD)	83.3 ± 3.1	82.5 ± 2.2	82.8 ± 2.4	0.194
Gender				0.081
Male	11 (36.7)	138 (53.5)	149 (51.7)	
Female	19 (63.3)	120 (46.5)	139 (48.3)	
BMI (kg/m^2^, mean ± SD)	24.4 ± 4.1	23.6 ± 3.6	23.9 ± 3.5	0.224
Distance from the AV				0.318
Lower (< 5 cm )	14 (46.7)	88 (34.1)	102 (35.4)	
Middle (5–10 cm )	9 (30.0)	111 (43.0)	120 (41.7)	
Upper (10–15 cm )	7 (23.3)	59 (22.9)	66 (22.9)	
ASA category				0.090
I–II	7 (23.3)	101 (39.1)	108 (37.5)	
III–IV	23 (76.7)	157 (60.9)	180 (62.5)	
Differentiation				0.565
Poor	8 (26.7)	56 (21.7)	64 (22.2)	
Moderate	18 (60.0)	179 (69.4)	197 (68.4)	
High	4 (13.3)	23 (8.9)	27 (9.4)	
Pathologic T stage				0.510
T1–T2	10 (33.3)	102 (39.5)	112 (38.9)	
T3–T4	20 (66.7)	156 (60.5)	176 (61.1)	
Pathologic *N* stage				0.642
N0	19 (63.3)	169 (65.5)	188 (65.3)	
N1	6 (20.0)	61 (23.6)	67 (23.3)	
N2	5 (16.7)	28 (10.9)	33 (11.4)	
Tumor size (cm, mean ± SD)	4.6 ± 1.9	4.4 ± 1.4	4.6 ± 1.6	0.663
Length of specimen removed (cm, mean ± SD)	10.5 ± 3.3	10.9 ± 2.7	10.5 ± 2.9	0.621
LN harvest (*n*, mean ± SD)	16.3 ± 7.4	17.2 ± 6.9	16.9 ± 7.3	0.733
Previous abdominal surgery	10 (33.3)	61 (23.6)	71 (24.7)	0.244

*BMI* body mass index, *ASA* American Society of Anesthesiologists, *AV* anal verge, *SD* standard deviation, *TNM stage* is based on the seventh edition of the American Joint Committee on Cancer (AJCC, the seventh edition) staging system

Tables 2 and 3 list the perioperative variables and surgical factors of the 330 patients, respectively. Among these patients, 138 (47.9%) underwent open AR or LAR, and 150 (52.1%) underwent laparoscopic AR or LAR. The mean duration of surgery was 143.6 ± 52.9 min, and the mean intraoperative blood loss was 84.4 ± 73.7 ml. There were 111 (38.5%) patients with three or more staples used for anastomosis. A total of 46 (16.0%) patients required a blood transfusion, and 36 (12.5%) patients had their left colic artery preserved. The mean time to first flatus was 5.5 ± 2.7 days, and the mean postoperative hospital stay was 11.6 ± 6.7 days. In addition, the patient comorbidities are shown in Table 4.

**Table 2:** Univariate analysis of perioperative variables

Factors	Anastomotic leakage (+) *n* = 30	Anastomotic leakage (−) *n* = 258	Total *n* = 288	*P*
Preoperative WBC (10^9^/L, mean ± SD)	5.8 ± 2.2	5.3 ± 1.8	5.6 ± 2.3	0.92
Preoperative PLT (10^9^/L, mean ± SD)	279.5 ± 110.7	252.4 ± 71.3	265 ± 98.8	0.833
Preoperative albumin (g/L, mean ± SD)	35.2 ± 4.6	38.9 ± 4.6	37.7 ± 4.4	0.067
Preoperative CRP (mg/L, mean ± SD)	0.63 ± 1.3	0.75 ± 1.1	0.69 ± 0.9	0.782
Preoperative HGB (g/L, mean ± SD)	122.9 ± 17.1	128.3 ± 17.6	124.9 ± 17.2	0.226
Neoadjuvant therapy	15 (50.0)	72 (27.9)	87 (30.2)	0.013
Postoperative application of albumin	16 (53.3)	110 (42.6)	126 (43.8)	0.264
Postoperative TPN starting time				0.158
≤ 24 h	16 (53.3)	103 (39.9)	119 (41.3)	
> 24 h	14 (46.7)	155 (60.1)	169 (58.7)	
Time to oral feeding (day, mean ± SD)	5.4 ± 2.3	5.4 ± 2.7	5.4 ± 2.5	0.915
Time to first flatus (day, mean ± SD)	5.8 ± 4.3	5.3 ± 2.3	5.5 ± 2.7	0.369
Postoperative hospital stay (day, mean ± SD)	20.3 ± 8.2	11.1 ± 4.6	11.6 ± 6.7	< 0.001

*WBC* white blood cell, *PLT* platelet, *CRP* c-reaction protein, *TPN* total parenteral nutrition, *SD* standard deviation

**Table 3:** Univariate analysis of surgical factors

Factors	Anastomotic leakage (+) *n* = 30	Anastomotic leakage (−) *n* = 258	Total *n* = 288	*P*
Types of operation				0.311
Open surgery	17 (56.7)	121 (46.9)	138 (47.9)	
Laparoscopic surgery	13 (43.3)	137 (53.1)	150 (52.1)	
Duration of operation (min, mean ± SD)	145.3 ± 67.1	141.4 ± 47.2	143.6 ± 52.9	0.452
Intraoperative blood loss (ml, mean ± SD)	109.1 ± 77.3	79.4 ± 70.5	84.4 ± 73.7	0.126
Number of stapler firings				0.011
< 3	12 (40.0)	165 (64.0)	177 (61.5)	
≥ 3	18 (60.0)	93 (36.0)	111 (38.5)	
Blood transfusion	7 (23.3)	39 (15.1)	46 (16.0)	0.368
Preservation of LCA	4 (13.3)	32 (12.4)	36 (12.5)	0.884
Temporary ileostomy	0 (0)	14 (5.4)	14 (4.9)	0.390

*LN* lymph node, *LCA* left colic artery, *SD* standard deviation

**Table 4:** Univariate analysis of comorbidity and others

Factors	Anastomotic leakage (+) *n* = 30	Anastomotic leakage (−) *n* = 258	Total *n* = 288	*P*
Any comorbidity	28 (93.3)	211 (81.8)	239 (83.0)	0.111
Diabetes	13 (43.3)	71 (27.5)	84 (29.2)	0.071
Hypertension	16 (53.3)	112 (43.4)	128 (44.4)	0.301
CHD	14 (46.7)	46 (17.8)	60 (20.8)	< 0.001
Arrhythmia	6 (20.0)	43 (16.7)	49 (17.0)	0.646
COPD	9 (30.0)	57 (22.1)	66 (22.9)	0.329
Hyperlipemia	10 (33.3)	98 (38.0)	108 (37.5)	0.618
Cerebrovascular disease	3 (10.0)	17 (6.6)	20 (6.9)	0.752
OAD	2 (6.7)	22 (8.5)	24 (8.3)	1.000
PVD	5 (16.7)	36 (14.0)	41 (14.2)	0.899
Viral hepatitis	3 (10.0)	31 (12.0)	34 (11.8)	0.980
Renal disease	5 (16.7)	44 (17.1)	49 (17.0)	0.957
Dementia	2 (6.7)	15 (5.8)	17 (5.9)	1.000
Immobility	2 (6.7)	14 (5.4)	16 (5.6)	1.000

### Univariate analysis of AL

We classified the patients into two groups based on whether they had AL, and the diagnostic criteria for AL were described above. Thirty patients met the diagnostic criteria for grades B and C and were diagnosed with AL, among which 21 patients met grade B criteria, and 9 patients met grade C criteria. We compared the two groups: 30 patients with AL versus 258 patients without AL. Univariate analysis showed that neoadjuvant therapy (50.0% vs. 27.9%, *P* = 0.013), number of stapler firings ≥ 3 (60.0% vs. 36.0%, *P* = 0.011), and CHD (46.7% vs. 17.8%, *P* < 0.001) were associated with an increased incidence of AL. In addition, male sex (36.7% vs. 53.5%, *P* = 0.081), preoperative albumin (35.2 vs. 38.9 g/ml, *P* = 0.067), and diabetes (43.3% vs. 27.5%, *P* = 0.071) tended to correlate with AL. The incidence of AL significantly prolonged the hospital stay (20.3 vs. 11.1 days, *P* < 0.001). Other factors were not significantly associated with AL.

### Multivariate logistic regression analysis of AL

The multivariate analysis showed that the number of stapler firings ≥ 3 (OR = 4.77, 95% CI = 1.33–15.21, *P* = 0.035), and CHD (OR = 8.33, 95% CI = 1.94–13.05, *P* = 0.003) were identified as independent risk factors for AL (Table 5).

**Table 5:** Multivariate analysis of AL

Factor	OR	95%CI	*P*
Neoadjuvant therapy	1.89	0.88–21.37	0.084
Number of stapler firings	4.77	1.33–15.21	0.035
CHD	8.33	1.94–13.05	0.003

*AL* anastomotic leakage, *OR* odds ratio, *95%CI* 95% confidence interval, *CHD* coronary heart diseases

## Discussion

AL remains one of the most dreaded complications after anterior resection for rectal cancer, with an incidence rate of approximately 10% reported in the literature [5, 6, 9, 10, 12–19]. This estimate includes asymptomatic anastomotic leakage, with an incidence as high as 50% [20]. In recent years, a large number of studies have reported risk factors associated with AL [5, 6, 9, 8–10]. Often, clinical characteristics such as gender, BMI, ASA, or sarcopenia have been assumed to be major contributors to the development of AL. With population aging, the incidence of rectal cancer in elderly patients shows an increasing trend. However, few relevant studies have confirmed whether elderly patients (age ≥ 80) with colorectal cancer have the same risk factors for anastomotic leakage as conventional patients with colorectal cancer. The purpose of the present study was to identify the risk factors associated with AL in elderly patients (age ≥ 80).

The use of more than two cutting closures is bound to increase the inclination, resulting in a weak area where the cutting line overlaps, which will increase the risk of AL [16–19]. Ito et al. [16] reported that the proportion of patients with vertical rectal division requiring three or more staples is smaller than that with transverse division (15% vs. 45%, *P* = 0.03), and multivariable analysis revealed that both TME (OR = 5.3; 95% CI = 1.2–22.7, *P* = 0.02) and three or more stapler firings during rectal division are significant and independent risk factors for AL (OR = 4.6; 95% CI = 1.1–19.2, *P* = 0.03). In 2009, Kim et al. [17] conducted a prospective study including 270 patients and reported that the application of multiple staples and anastomotic levels are major contributors to the development of AL. Park et al. [18] also provided strong evidence that the number of linear stapler firings > 3 increased the risk of AL after laparoscopic surgery for rectal cancer in 2013 (OR = 7.849; 95% CI = 3.776–16.314, *P* < 0.001). In our study, there were 111 (38.5%) patients with three or more staples for anastomosis; among them, 18 had AL and 93 had no AL. Univariate analysis showed that the number of stapler firings ≥ 3 was significantly different between the two groups (60.0% vs. 36.0%, *P* = 0.011). Multivariate logistic regression analysis showed that the number of stapler firings ≥ 3 was an independent risk factor for AL (OR = 4.77, 95% CI = 1.33–15.21, *P* = 0.035). These results are consistent with the results mentioned above. The increased use of stapler firings is often associated with some unfavorable conditions, such as low rectal cancer in obese male patients, patients with vascular disease after radiotherapy, or patients with previous multiple abdominopelvic operations.

Elderly patients have more comorbidities than young patients do. Multivariate logistic regression analysis in the present study showed that CHD was an independent risk factor for AL (OR = 8.33, 95% CI = 1.94–13.05, *P* = 0.003). How coronary heart disease affects the occurrence of AL has not been determined conclusively. A basic study by Fawcett found that the intestinal serosal microcirculation blood supply was an important factor for anastomotic healing [21]. Laser-Doppler flowmetry showed that the decrease in blood flow signal at the rectal stump was closely related to the occurrence of anastomotic leakage [22]. CHD is generally associated with systemic atherosclerosis that includes the blood vessels of the rectal stump [23], and the microcirculation blood supply condition of the rectal stump directly determines anastomotic healing. This notion remains to be investigated by a pathophysiological study. Furthermore, a prospective study including 276 rectal cancer patients conducted by Kruschewski et al. [24] showed that smokers had an increased risk of anastomotic leakage [OR = 6.42; 95% CI = 2.68–15.36], as did patients with coronary heart disease (OR = 7.79; 95% CI = 2.52–24.08). Our results were consistent with these findings.

A large number of studies have reported that the incidence of AL after anterior rectal resection was significantly increased by various factors, such as male sex, diabetes, and low tumor position. Our study similarly found that male sex (36.7% vs. 53.5%, *P* = 0.081) and diabetes (43.3% vs. 27.5%, *P* = 0.071) tended to be correlated with the occurrence of AL in elderly patients, which were comparable to those findings in young patients. Moreover, we also found that the number of stapler firings ≥ 3 and CHD were independent risk factors for AL in elderly patients (age ≥ 80) with rectal cancer. The limitations of this study include its retrospective nature and the relatively small patient sample size. In addition, the present study lacked an analysis of the effect of sarcopenia on AL after anterior resection of rectal cancer in the elderly. Multicenter large-scale prospective studies are worth pursuing to verify our results.

## Conclusion

The number of stapler firings ≥ 3 and CHD were independent risk factors for AL in elderly patients (age ≥ 80) with rectal cancer. A temporary ileostomy or the Hartmann procedure is recommended for male patients and patients with CHD, obesity, or lower tumor location, which may increase the number of stapler firings used. Certainly, we recommend that the number of stapler firing should be minimized to alleviate the economic and physical burden of patients.

## Data Availability

All data generated or analyzed during this study are included in this published article.
